# Supplementary material for: Nifedipine Improves the Ketogenic Diet Effect on Insulin-Resistance-Induced Cognitive Dysfunction in Rats
Source: Pharmaceuticals (Basel). 2024 Aug 10;17(8):1054. doi: 10.3390/ph17081054 (PMC11359371; doi:10.3390/ph17081054)
Supplement: Supplementary file 1 [file pharmaceuticals-17-01054-s001.zip › pharmaceuticals-3117693-supplementary.pdf]

Animal weight

Normal rats	IR+ Normal diet	IR+Ketogenic diet	IR+Normal Diet + Nifedipine	IR+ ketognic diet +Nifedipine
11.55	-2.89	8.4	-11.91	-6.66
14.38	-5.22	4.86	-14.85	2.49
15.6	-27.76	8.12	-10.5	3.85
11.5	-3.53	5.45	-10.07	0.73
18.56	-4.37	9.65	-12.59	-6
15.83	-7.71	7.23	-11.72	21.36

OGTT

Time	Non-treated with fructose 10 %						
0	82	84	85	83	85	84	132
30	151	156	145	139	137	138	151
60	161	149	147	163	154	153	237
90	126	144	118	127	129	116	171

Glucose

Normal rats	IR+ Normal diet	IR+Ketogenic diet	IR+Normal Diet + Nifedipine	IR+ ketognic diet +Nifedipine
70	68	90	87	94
74	88	93	94	86
66	75	73	70	99
70	75	85	84	93
70	77	80	91	96
71	82	87	82	90

Insulin

Normal rats	IR+ Normal diet	IR+Ketogenic diet	IR+Normal Diet + Nifedipine	IR+ ketognic diet +Nifedipine
7.2	7.4	9.8	9.8	10.3
8.5	9.7	13.3	10.3	9.4
7.4	8.2	8.7	8.5	11.2
7.7	8.6	10.5	9.5	10.3
8	9.2	10.1	9	9.9
7.5	8.2	11.2	10.1	10.8

HOMA-IR index

Normal rats	IR+ Normal diet	IR+Ketogenic diet	IR+Normal Diet + Nifedipine	IR+ ketognic diet +Nifedipine
1.33	1.24	2.1	2.11	2.39

1.56	2.1	3.1	2.39	1.99
1.17	1.54	1.57	1.47	2.73
1.35	1.63	2.26	1.99	2.55
1.45	1.39	1.84	1.73	2.37
1.25	1.75	2.68	2.25	2.19

Cholesterol

Normal rats	IR+ Normal diet	IR+Ketogenic diet	IR+Normal Diet + Nifedipine	IR+ ketognic diet +Nifedipine
59	60	80	78	82
63	67	80	83	77
58	65	74	69	86
59	63	75	77	82
59	65	74	81	80
61	65	75	73	84

T.G

Normal rats	IR+ Normal diet	IR+Ketogenic diet	IR+Normal Diet + Nifedipine	IR+ ketognic diet +Nifedipine
48	50	67	67	71
52	55	70	70	68
47	52	61	60	75
48	52	68	66	71
50	56	69	69	70
48	52	66	63	73

LDL

Normal rats	IR+ Normal diet	IR+Ketogenic diet	IR+Normal Diet + Nifedipine	IR+ ketognic diet +Nifedipine
20	25	42	32	31
22	24	49	34	28
21	23	46	29	29
20	23	44	32	29
21	24	44	33	29
21	22	47	31	30

HDL

Normal rats	IR+ Normal diet	IR+Ketogenic diet	IR+Normal Diet + Nifedipine	IR+ ketognic diet +Nifedipine
40	37	28	28	28
36	36	27	26	32
42	33	29	30	31

41	34	28	28	30
39	36	29	29	29
41	33	26	27	32

BDNF

Normal rats	IR+ Normal diet	IR+Ketogenic diet	IR+Normal Diet + Nifedipine	IR+ ketognic diet +Nifedipine
43	41	32	31	30
93	39	31	30	28
46	50	33	34	28
61	45	32	32	29
76	49	31	33	30
43	39	32	34	29

GSK3β

Normal rats	IR+ Normal diet	IR+Ketogenic diet	IR+Normal Diet + Nifedipine	IR+ ketognic diet +Nifedipine
10	10	14	33	24
9	11	20	30	21
14	10	16	27	23
12	10	19	30	22
11	14	19	29	23
11	12	16	32	24

IDE

Normal rats	IR+ Normal diet	IR+Ketogenic diet	IR+Normal Diet + Nifedipine	IR+ ketognic diet +Nifedipine
37	37	28	26	25
36	34	26	25	23
40	44	26	30	26
39	40	27	27	25
39	42	26	28	24
38	39	29	26	26

Aβ Cerebral cortex

Normal rats	IR+ Normal diet	IR+Ketogenic diet	IR+Normal Diet + Nifedipine	IR+ ketognic diet +Nifedipine
6	92	36	80	10
6	93	36	90	15
9	86	43	95	25
5	89	40	88	20
7	89	37	85	16

6	91	37	89	18

Aβ hippocampus

Normal rats	IR+ Normal diet	IR+Ketogenic diet	IR+Normal Diet + Nifedipine	IR+ ketognic diet +Nifedipine
4	92	36	60	10
4	93	36	55	15
10	87	44	70	25
6	88	39	60	15
10	88	44	65	10
6	87	36	65	20

Tau protein cerebral cortex

Normal rats	IR+ Normal diet	IR+Ketogenic diet	IR+Normal Diet + Nifedipine	IR+ ketognic diet +Nifedipine
13	44	23	40	21
11	43	23	39	17
9	39	18	35	22
12	45	19	38	20
13	44	22	37	18
10	45	22	38	19

Tau protein hippocampus

Normal rats	IR+ Normal diet	IR+Ketogenic diet	IR+Normal Diet + Nifedipine	IR+ ketognic diet +Nifedipine
14	44	68	40	21
11	41	62	39	17
11	39	55	35	22
14	40	62	38	20
11	41	63	37	23
14	44	68	34	19

Normal rats

Day 1	30.75	34.5	24.75	24.25	17.5	24.75	40.25
Day 2	10.75	5.25	24.25	17.25	32	27.75	6.75
Day 3	9.25	7.5	9.25	4.75	10.5	7.25	5.75
Day 4	14.5	6	11	12.75	16.5	9	8
Day 5	34	55	41	44	34	29	20

Treated with fructose 10%

111	113	115	117	122
190	172	161	240	204
206	234	240	334	165
200	234	221	353	150

Moris water maze test

	IR+ Normal diet						IR+Ketogenic diet					IR+Normal Diet + Nifedi		
52	82.75	25.75	72.5	98	34	83.75	85.25	43.75	50	90	49.5	36.75	39.25	45.75
10.5	29.75	37.75	17.5	40.5	16.5	19.25	21.5	13.55	14.75	15.8	8.75	8.25	6.75	6.75
8.5	6.5	6	11	12	6.5	10	6.5	4.75	5	3.25	4	4.25	6.5	13
3.25	9.75	5.75	4.25	11.5	5.5	13	5.25	4.75	3.215	3.5	6.25	6.75	5.25	3
20	43	44	40	45	63	66	34	48	50	48	49	38	47	48

pine			IR+ ketognic diet +Nifedipine				
45.75	59.25	60.75	43	82.5	50.75	42	53.5
9.25	20.5	13	19.5	46.25	13.25	6	17.75
7.25	4.25	3	6.5	25.75	11	7	6.5
2.25	3.75	5	3.25	3.75	6.25	3	3.75
56	45	55	42	26	50	37	45
